# Novel discovery of exosomes biomarker from herpes zoster viral infection: linking to cerebrovascular coincidence

**DOI:** 10.1097/MS9.0000000000000220

**Published:** 2023-02-17

**Authors:** Ruhul Amin, Kuldeep Dhama, Talha B. Emran

**Affiliations:** a Faculty of Pharmaceutical Science, Assam Down Town University, Panikhaiti, Guwahati, Assam, India; b Division of Pathology, ICAR-Indian Veterinary Research Institute, Bareilly, Izatnagar, Uttar Pradesh, India; c Department of Pharmacy, BGC Trust University Bangladesh, Chittagong, Bangladesh; d Department of Pharmacy, Faculty of Allied Health Sciences, Daffodil International University, Dhaka, Bangladesh

The herpes virus known as varicella-zoster virus (VZV) is one of eight that may infect humans. Varicella (commonly known as ‘chickenpox’) and herpes zoster (shingles) are two separate diseases caused by VZV infection (shingles). Chickenpox, or the VZV rash, is a systemic inflammatory response to an initial VZV infection. Herpes zoster, often known as a zoster virus infection, is a skin illness caused by the endogenous reactivation of latent VZV.

Ischemic stroke, in which blood flow to the brain is cutoff because of constricted arteries or a clot, is among the most serious neurological disorders. Stroke risk is around 80% greater in those with shingles than in those without the illness and remains elevated for up to a year after the rash has cleared^1^.

It is unclear what mechanisms contribute to this increased stroke risk over time. It has been hypothesized by some experts that this is due to an infection of the arteries themselves^2^. Although this may be the case in certain cases, several aspects of VZV infections indicate that this is not the whole story. A hallmark of VZV infections is the development of systemic inflammation that may last for weeks or months after the virus is no longer visible and is thought to be dormant again^3^. A recently published study discovered that VZV reactivation causes cells to release sacks called exosomes that are laden with proteins that aid in blood clotting and inflammation^4^. It is possible that having more of these proteins in your blood might raise your chance of having a stroke.

Nucleic acids, proteins, lipids, and metabolites are all transported in exosomes, which are extracellular vesicles produced by all cells. They play an important role in both healthy and diseased cells as messengers of short- and long-distance intercellular communication. Having shingles raises your chance of having a stroke because exosomes produced by your body contain proteins involved in blood clotting. A study compared exosomes extracted from the blood of 13 individuals with a shingles outbreak to those isolated from the blood of healthy volunteers^4^. Examination of the exosome composition revealed that those with shingles had nine times more clotting proteins than those without the disease.

Further, they discovered that 3 months after the first rash, the exosomes of shingles patients still exhibited higher quantities of these proteins. By exposing healthy platelets to exosomes from shingles patients or healthy individuals, we were able to establish that the contents of these exosomes may cause clotting. When platelets were exposed to shingles exosomes, they began to combine with other blood cells, much as they would during the clotting process.

Shingrix, a vaccine licensed by the Food and Drug Administration to prevent shingles, is now accessible to individuals 50 and older and to persons with immunosuppression aged 18 and older^5^. However, persons under the age of 40 who are at the greatest risk of stroke are ineligible for Shingrix^6^.

While this study offers evidence for a putative mechanism through which shingles might raise the risk of stroke during and shortly after infection, further work is required to determine the duration of this risk.

Exosomes’ potential as a biomarker for tracking stroke risk after shingles will also be investigated in these long-term trials. Meanwhile, we are hoping that our paper will inspire more individuals to obtain the shingles vaccine and serve as a possible target for future drug research.

## Ethical approval

This case report has been reported in line with the Case Report (CARE) guidelines.

## Consent

Not applicable.

## Sources of funding

This study received no specific grant from any funding agency in the public.

## Author contribution

R.A.: conceptualization, data curation, writing – original draft preparation, writing – reviewing and editing. K.D.: data curation, writing – original draft preparation, writing – reviewing and editing. T.B.E.: writing – reviewing and editing, visualization, supervision.

## Conflicts of interest disclosure

The authors have read and understood the policy on declaration of interests and have no relevant interests to declare. The responsibility for the content lies with the author and the views stated herein should not be taken to represent those of any organizations or groups with and for which he works. All authors report no conflicts of interest relevant to this article.

## Research registration unique identifying number (UIN)

1. Name of the registry: not applicable.

2. Unique identifying number or registration ID: not applicable.

3. Hyperlink to your specific registration (must be publicly accessible and will be checked): not applicable.

## Guarantor

Talha Bin Emran, PhD, Associate Professor, Department of Pharmacy, BGC Trust University Bangladesh, Chittagong 4381, Bangladesh. Tel: +880 303 356 193, Fax: +880 312 550 224. https://orcid.org/0000-0003-3188-2272. E-mail: talhabmb@bgctub.ac.bd

## Provenance and peer review

Not commissioned, externally peer reviewed.
